# Natural ursolic acid based self-therapeutic polymer as nanocarrier to deliver natural resveratrol for natural therapy of acute kidney injury

**DOI:** 10.1186/s12951-023-02254-x

**Published:** 2023-12-17

**Authors:** Yuanpeng Nie, Liying Wang, Shengbo Liu, Chunlei Dai, Tianjiao Cui, Yan Lei, Xinru You, Xiaohua Wang, Jun Wu, Zhihua Zheng

**Affiliations:** 1 Department of Nephrology, Center of Kidney and Urology, The Seventh Affiliated Hospital, Sun Yat-Sen University, Shenzhen, 518107 China; 2 Department of Hematology, The Seventh Affiliated Hospital, Sun Yat-Sen University, Shenzhen, 518107 China; 3 Department of Thoracic Surgery, Guangdong Provincial People’s Hospital (Guangdong Academy of Medical Sciences), Southern Medical University, Guangzhou, 510080 China; 4 School of Biomedical Engineering, Sun Yat-Sen University, Shenzhen, 518107 China; 5 Center for Nanomedicine and Department of Anesthesiology, Brigham and Women’s Hospital, Harvard Medical School, Boston, MA 02115 USA; 6 Bioscience and Biomedical Engineering Thrust, The Hong Kong University of Science and Technology (Guangzhou), Nansha, Guangzhou, 511400 China; 7 Division of Life Science, The Hong Kong University of Science and Technology, Hong Kong SAR, China

**Keywords:** Poly(ursolic acid), Resveratrol, Acute kidney injury, Drug delivery

## Abstract

Acute kidney injury (AKI) is a common kidney disease associated with excessive reactive oxygen species (ROS). Unfortunately, due to the low kidney targeting and undesired side effects, the existing antioxidant and anti-inflammatory drugs are unavailable for AKI management in clinic. Therefore, it’s essential to develop effective nanodrugs with high renal targeting and biocompatibility for AKI treatment. Herein, we reported a novel nanodrug for AKI treatment, utilizing poly(ursolic acid) (PUA) as a bioactive nanocarrier and resveratrol (RES) as a model drug. The PUA polymer was synthesized form ursolic acid with intrinsic antioxidant and anti-inflammatory activities, and successfully encapsulated RES through a nanoprecipitation method. Subsequently, we systemically investigated the therapeutic potential of RES-loaded PUA nanoparticles (PUA NPs@RES) against AKI. In vitro results demonstrated that PUA NPs@RES effectively scavenged ROS and provided substantial protection against H_2_O_2_-induced cellular damage. In vivo studies revealed that PUA NPs significantly improved drug accumulation in the kidneys and exhibited favorable biocompatibility. Furthermore, PUA NPs alone exhibited additional anti-inflammatory and antioxidant effect, synergistically enhancing therapeutic efficacy in AKI mouse models when combined with RES. Overall, our study successfully developed an effective nanodrug using self-therapeutic nanocarriers, presenting a promising option for the treatment of AKI.

**Graphical abstract:**

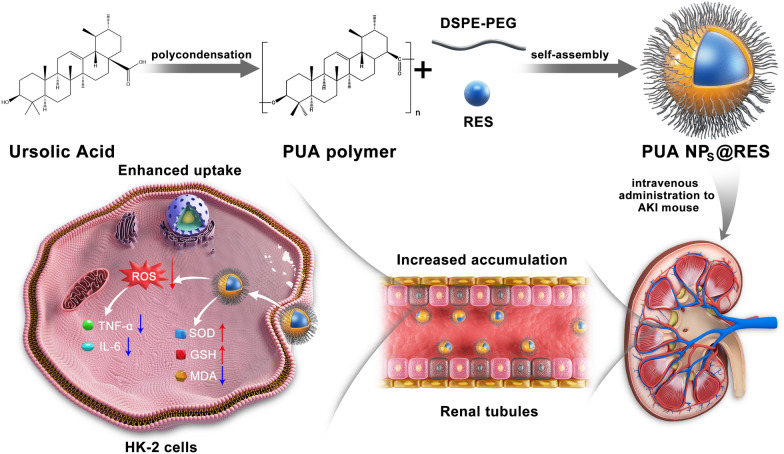

**Supplementary Information:**

The online version contains supplementary material available at 10.1186/s12951-023-02254-x.

## Introduction

Acute kidney injury (AKI) is a common kidney disease characterized by a sudden and rapid decline in renal function over a short period [1]. The incidence of AKI has greatly increased in recent years, attributed to factors such as an aging population and changing lifestyles [2]. However, diagnosing AKI accurately remains challenging due to the absence of a definitive gold standard, which subsequently poses difficulties in its treatment [3]. Currently, treatment options for AKI are limited to supportive care [4]. Therefore, there is an urgent need to develop new and effective strategies for AKI treatment.

Reportedly, excessive reactive oxygen species (ROS) play an important role in the progression of AKI [5, 6]. ROS can induce oxidative stress in cells, leading to cell death and activating various pathways associated with lysosome damage, inflammation, apoptosis, and necrosis. These processes can eventually result in acute tubular necrosis and renal failure [7]. Consequently, scavenging ROS presents a promising strategy for AKI treatment. Some small molecule antioxidants, such as *N*-acetylcysteine, curcumin and polyphenol, have been used for AKI treatment [8–11]. However, their efficacy has been limited by poor renal targeting and undesired side effects [12–14]. Fortunately, the development of AKI nanodrugs offers a potential solution to address these challenges. These nanodrugs possess distinct advantages, such as improved renal targeting and enhanced biosafety, owing to their unique size and various surface modification strategies [15–17]. While many AKI nanodrugs focus on purely delivering antioxidant and anti-inflammatory drugs to the kidney, there is an opportunity to further enhance their therapeutic effects by incorporating self-therapeutic nanocarriers. By leveraging these carriers, a synergistic therapeutic effect can be achieved, thereby aiming to enhance effectiveness and reduce toxicity [18].

Ursolic acid (UA), a natural triterpene compound derived from various plants, has gained significant attention in recent years for its potential in treating cancer and ROS-related diseases. This is attributed to its diverse pharmacological properties, including antioxidant, anti-inflammatory, anticancer, and hepatoprotective effects [19–21]. In our previous study, we innovatively utilized UA as a monomer to synthesize a bioactive polymer called poly(ursolic acid) (PUA). By leveraging the inherent anti-cancer activity of UA, the PUA polymer served as a nanocarrier, exhibiting synergistic therapeutic effects when combined with an anticancer drug [22].

**Scheme.1:**
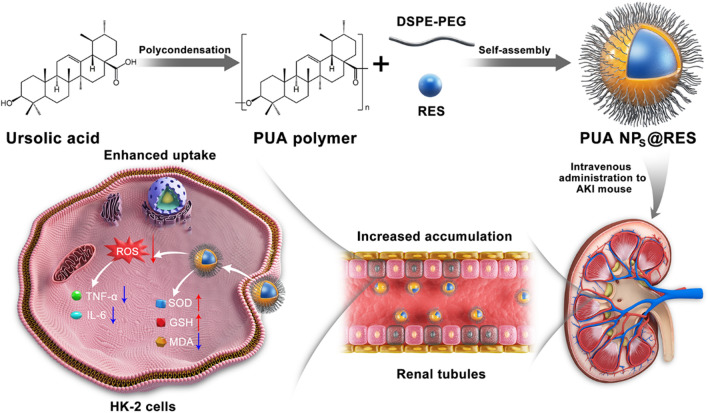
Illustration showing the construction of PUA NPs@RES and the therapeutic mechanism for alleviating acute kidney injury

Inspired by our previous findings and considering the antioxidant and anti-inflammatory activities of UA, we expanded the application of PUA in the treatment of AKI by utilizing it as a self-therapeutic nanocarrier for the targeted delivery of antioxidant drugs. In this study, a natural antioxidant, resveratrol (RES), was selected as a model drug. RES is a non-flavonoid polyphenol organic compound produced by plants, and is well known as "natural medicine" due to its antioxidant, anti-inflammatory, anti-aging, cardiovascular protective effects. However, the application of RES has been hindered by its limited water solubility and bioavailability [23–25]. To improve the therapeutic potential of RES in AKI therapy, RES was encapsulated into the PUA polymer through a nanoprecipitation method, forming RES-loaded PUA nanoparticles (PUA NPs@RES) (Scheme 1). Subsequently, we systemically explored the therapeutic potential of PUA NPs@RES against AKI. In vitro studies demonstrated the superior ROS-scavenging activity of PUA NPs@RES from various aspects. In vivo therapeutic efficacy of PUA NPs and PUA NPs@RES were further assessed in glycerol-induced AKI mouse models. More importantly, the therapeutic mechanism underling the PUA nanocarrier was revealed by sequencing analysis for the first time.

## Results and discussion

### Synthetization and characterization of PUA polymer

**Fig. 1:**
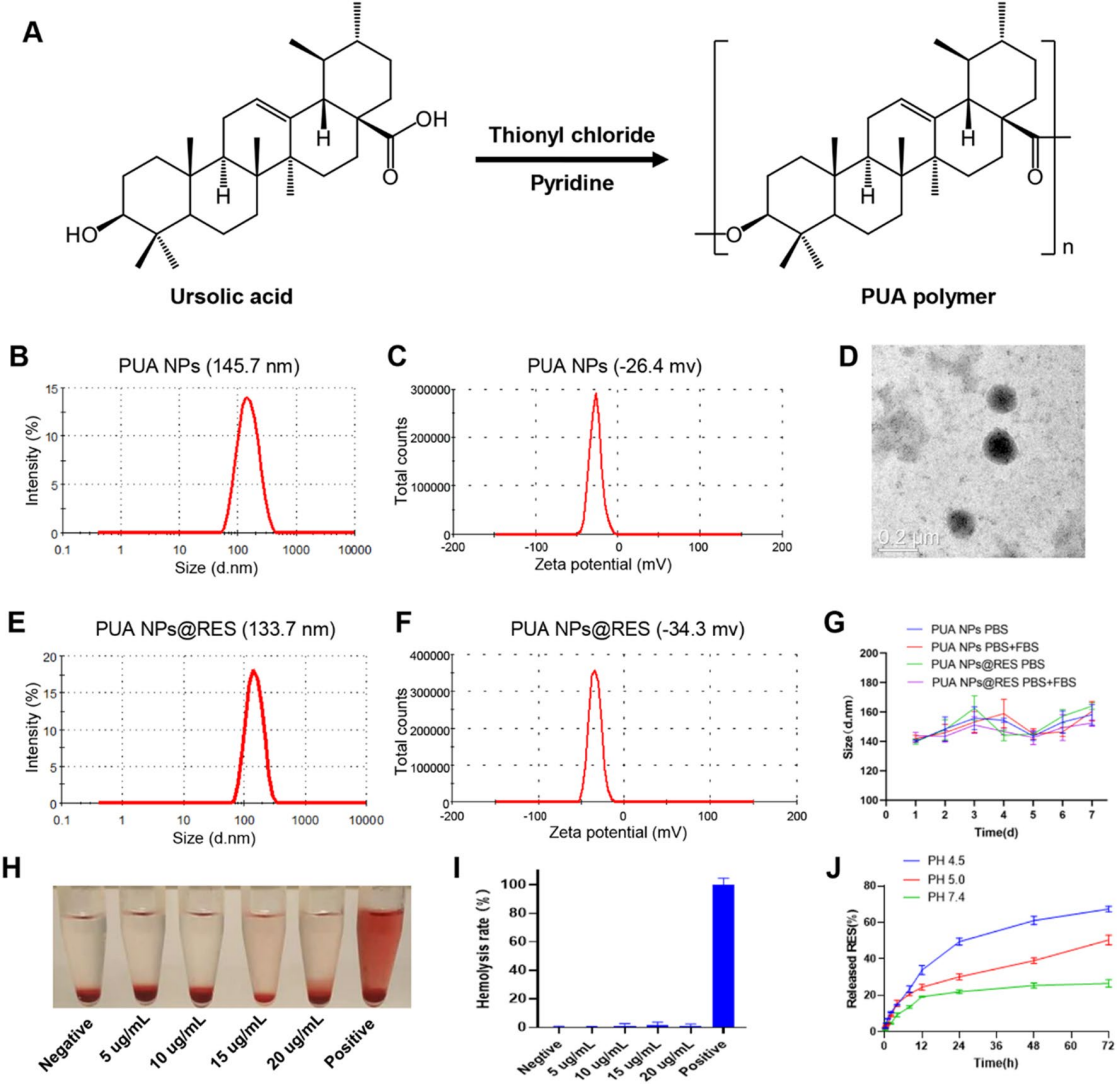
Preparation and characterization of the NPs. **A** Synthetic route of the PUA polymer. **B** DLS size distribution and **C** zeta potential of the PUA NPs. and **C** PUA NPs@RES. **D** Representative TEM images of the PUA NPs@RES. Scale bar = 200 nm. **E** DLS size distribution and **F** zeta potential of the PUA NPs@RES. **G** The particle size changes of PUA NPs and PUA NPs@RES in different media for 7 days **H** Representative images of red blood cells after treatment with various concentrations of PUA NPs@RES, water (positive), and saline solution (negative). **I** Quantitative analysis of hemolysis ratio. **J** In vitro drug release profiles of PUA NPs@RES in media with different pH values

The PUA polymer was synthesized using a simple and rapid polycondensation method with UA as the monomer, as previously reported [26] (Fig. 1A). The polycondensation of carboxyl and hydroxyl groups between UA was confirmed by ^1^H-nuclear magnetic resonance (^1^H-NMR) results, as shown in Additional file 1: Fig. S1 and S2. In addition, Fourier transform infrared spectroscopy (FT-IR) was used to further verify the structure of PUA. As shown in Additional file 1: Fig. S3, the spectrum of PUA exhibits a characteristic band of ester bond at 1762 cm^−1^, while the stretching vibration peak of hydroxyl group at 3444 cm^−1^ disappears, further confirming the successful synthesis of PUA. Gel permeation chromatography (GPC) measurement revealed that the average molecular weight (Mw) of PUA was 2457 Da (Additional file 1: Table S1).

### Preparation and characterization of PUA NPs and PUA NPs@RES

The generated PUA polymer is hydrophobic but can be dissolved in organic solvents such as dimethyl sulfoxide (DMSO). Thereby, PUA NPs were prepared by nanoprecipitation method with DSPE-PEG 2 K as stabilizer. Hydrophobic PUA and amphiphilic DSPE-PEG 2 K can be self-assembled in water to form stable and small-sized NPs. PUA NPs loaded with RES (PUA NPs@RES) were prepared using the same method with the addition of RES.

We prepared a series of PUA NPs@RES with different mass ratios of RES and PUA. When the ratio of PUA to RES was 3:1, the obtained PUA NPs@RES exhibited maximal drug loading capacity and good stability, and were therefore selected for subsequent studies. (Additional file 1: Table S2). Compared with PUA NPs with a diameter of ~ 145.7 nm, a PDI of 0.229, and a zeta potential of -26.4 mV (Fig. 1B and C), PUA NPs@RES slightly differ in these parameters, showing a smaller size of ~ 133.7 nm, a PDI of 0.221 and a zeta potential of − 34.3 mV (Fig. 1E and F). The difference in particle size might be attributed to the hydrophobic interactions between RES and PUA polymer, which facilitate the formation of a more compact core within PUA NPs@RES, resulting a smaller particle size. Transmission electron microscopy (TEM) imaging further confirmed the size and spheroidal morphology of PUA NPs and PUA NPs@RES (Fig. 1D and Additional file 1: Fig S4). In addition, we evaluated the in vitro stability of PUA NPs and PUA NPs@RES over an extended period by monitoring changes in size in different solutions. The results showed that the size of PUA NPs and PUA NPs@RES remained basically unchanged in either PBS alone or PBS containing 10% serum within 7 days (Fig. 1G). Figure 1H shows hemolysis ratios of PUA NPs@RES at different RES concentrations. Obviously, after treatment with PUA NPs@RES, the supernatant of erythrocyte suspension after centrifugation was colorless and transparent, with no erythrocyte rupture observed. Hemolysis rates of PUA NPs@RES across different concentrations were all < 5% (Fig. 1I). Taken together, PUA NPs@RES possessed good blood compatibility, which is promising for their future In vivo application.

### Drug release behavior of PUA NPs@RES

Next, we studied the drug release rate of PUA NPs@RES under varying pH conditions. The pH values ranged from pH 7.4 to 4.5 to simulate the normal environment and acidic microenvironment of AKI. As shown in Fig. 1J, PUA NPs@RES exhibited different release curves with pH changing. After 24 h, the cumulative release of RES reached 22.0% in the medium with pH 7.4, while that of RES significantly to 30.2% and 49.5% under pH of 5.0 and 4.5, respectively. After 72 h, the cumulative release of RES in normal environment slightly increased to 26.6%. However, under acidic environments with pH 5.0 and 4.5, the cumulative release reached 50.5% and 67.5%, respectively. This may be attributed to the increased breaking of the ester bonds of PUA in the acidic environment, which leads to the destruction of the structure of PUA NPs@RES and ultimately increases release of RES.

### Cell uptake of PUA NPs@RES

**Fig.2:**
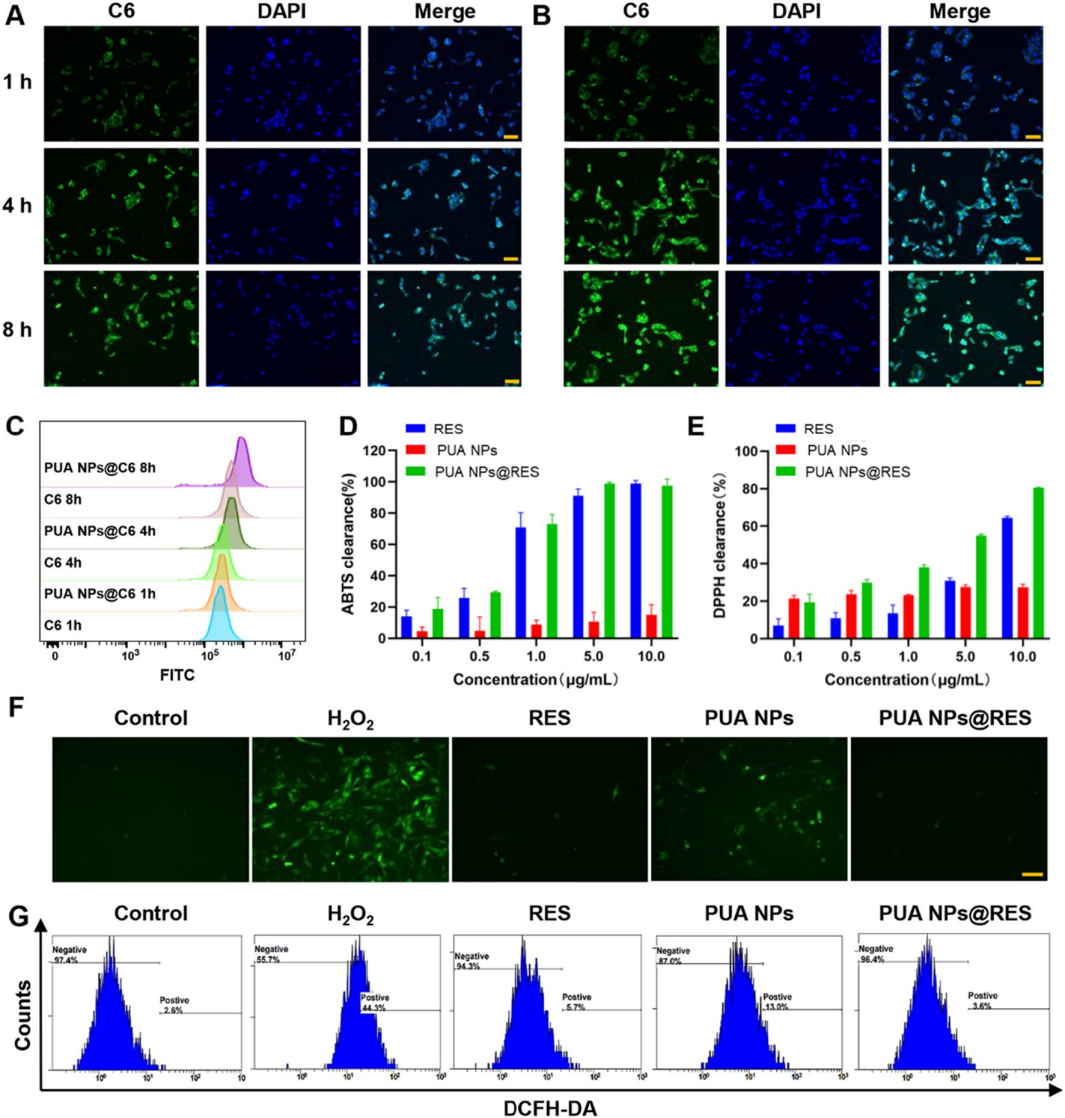
Evaluation of cellular uptake and antioxidative performance in vitro. Cellular uptake images of H_2_O_2_-stimulated HK-2 cells incubated with **A** coumarin 6 and **B** PUA NPs@C6 NPs for 1, 4, and 8 h, respectively. Scale bar: 100 μm. **C** Flow cytometry analysis of cellular uptake. **D** ABTS· and **E** DPPH· scavenging efficiency of RES, PUA NPs and PUA NPs@RES across various equivalent RES concentrations. **F** Fluorescence images of intracellular ROS levels of H_2_O_2_-stimulated HK-2 cells measured by DCFH-DA assay after different treatments. Scale bar: 100 μm. **G** Flow cytometry analysis of intracellular ROS after different treatments

Cytotoxicity test was performed to assess the toxicity of PUA NPs and PUA NPs@RES on Human kidney-2 (HK-2) cells. The results showed that the survival rate of HK-2 cells in both PUA NPs and PUA NPs@RES was higher when RES concentration was less than 10 ug/ml, indicating low toxicity of NPs at low concentrations (Additional file 1: Fig. S5). We then prepared coumarin-6 (C6) loaded PUA NPs (PUA NPs@C6) and investigated the cellular uptake of NPs on HK-2 cells. The cells were first treated with H_2_O_2_ to induce activation, and then incubated with free C6 and PUA NPs@C6 for 1, 4 and 8 h. As shown in Fig. 2A and B, the fluorescence intensity of free C6 and PUA NPs@C6 both enhanced over time, with PUA NPs@C6 exhibiting stronger fluorescence at 4 and 8 h. Next, the cellular uptake was quantitively confirmed by flow cytometry analysis (Fig. 2C and Additional file 1: Figure: S6), further indicating the increased internalization of PUA NPs@C6 by HK-2 cells.

### In vitro antioxidant properties of PUA NPs@RES

The kidneys are susceptible to ROS damage due to their abundant blood supply [7]. An increase in ROS can be particularly harmful to the renal tubules, ultimately leading to AKI [27]. Therefore, we first investigated the antioxidant properties of PUA NPs@RES and its ability to scavenge free radicals, as measured by ABTS· and DPPH· scavenging assays (Fig. 2D and E). The results demonstrated that PUA NPs@RES could effectively scavenge different free radicals in a concentration and time dependent manner, which was primarily beneficial from the intrinsic antioxidant properties of loaded RES. Meanwhile, it is worth noting that PUA carrier itself also exhibited certain free radical scavenging ability.

To evaluate whether PUA NPs@RES can protect HK-2 cells from H_2_O_2_-induced oxidative stress, ROS staining was performed on HK-2 cells to observe the fluorescence intensity of dichlorodihydrofluorescein diacetate (DCFH-DA) probe in the cells. As shown in Fig. 2F, strong fluorescence was observed in cells stimulated with H_2_O_2,_ indicating high intracellular ROS production. In contrast, both RES and PUA NPs@RES treatments significantly reduced intracellular ROS levels. Additionally, PUA NPs treatment also partially scavenged excess ROS in cells. Flow cytometry analysis further confirmed that RES, PUA NPs and PUA NPs@RES can alleviate H_2_O_2_-induced oxidative stress. (Fig. 2G and Additional file 1: S7).

**Fig. 3:**
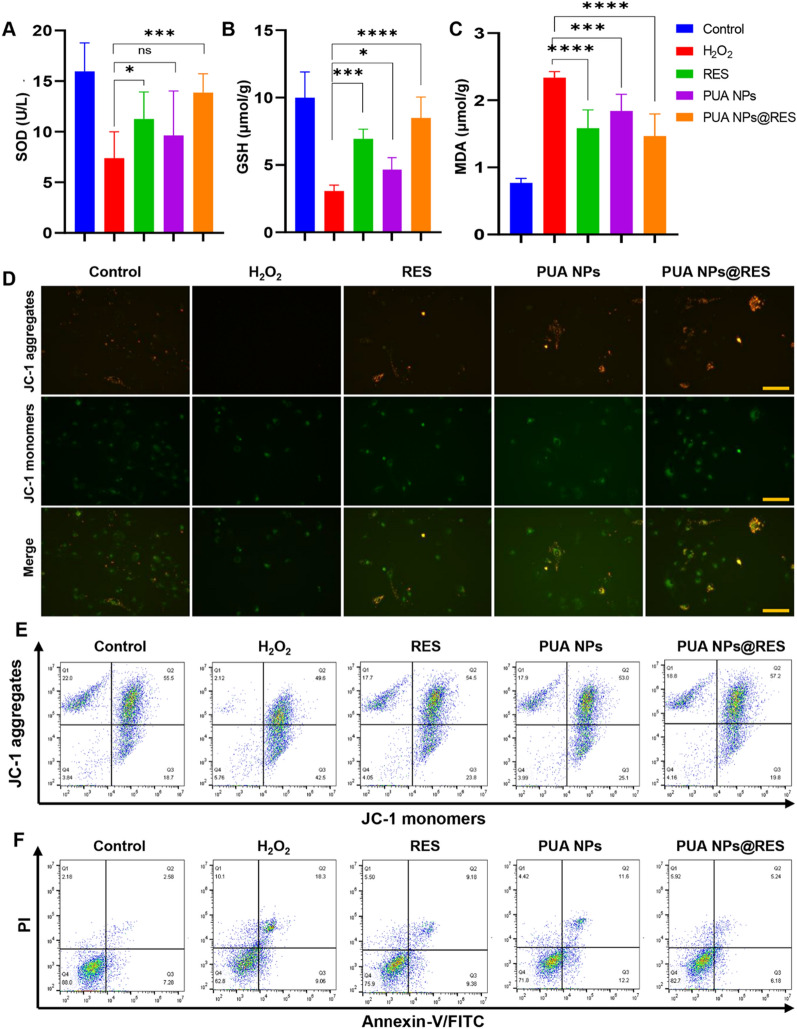
Evaluation of antioxidant and anti-apoptotic activity of PUA NPs@RES in vitro. **A** SOD level, **B** GSH level and **C** MDA level of H_2_O_2_-stimulated HK-2 cells after treatment with PUA NPs, RES, and PUA NPs@RES at an equivalent RES concentration of 5 μg/mL for 24 h. **D** Mitochondrial membrane potential of H_2_O_2_-stimulated HK-2 cells treated with PUA NPs, RES, and PUA NPs@RES at an equivalent RES concentration of 5 μg/ml for 24 h. Scale bar: 100 μm. **E** Flow cytometry analysis of mitochondrial membrane potential of H_2_O_2_-stimulated HK-2 cells after treatments for 24 h. **F** Cell apoptosis of H_2_O_2_-stimulated HK-2 cells after different treatments

Intracellular superoxide dismutase (SOD) and glutathione (GSH) are pivotal in maintaining the cellular redox balance by scavenging ROS, which can also reflect intracellular antioxidant status. We therefore assessed the intracellular SOD and GSH levels in HK-2 cells treated with different formulations (Fig. 3A and B). We found that treatment with H_2_O_2_ significant reduced SOD and GSH levels in the cells, whereas RES, PUA NPs and PUA NPs@RES halted this decrease to different extents, suggesting their capability to effectively protect cells against H_2_O_2_-induced oxidative stress. Remarkably, PUA NPs@RES exerted the most potent protection. Additionally, evaluation of intracellular Malonaldehyde (MDA), which reflects lipid peroxidation levels, further demonstrated that RES, PUA NPs, and PUA NPs@RES can protect cells from lipid peroxidation damage by clearing ROS (Fig. 3C). Taken together, the antioxidative properties of RES and nanocarriers enabled PUA NPs@RES to restore intracellular antioxidant capacity, making it a promising AKI treatment candidate.

### Cell apoptosis analysis

Mitochondrial membrane potential (MMP) is a critical indicator of mitochondrial function and can be used for early apoptosis detection [28]. The changes in MMP can be reflected by fluorescence staining of JC-1 probe. Specifically, when the MMP decreased, the fluorescence of JC-1 changed from red to green. Therefore, after H_2_O_2_ treatment, the red fluorescence of HK-2 cells weakened, while the green fluorescence enhanced. In contrast, treatment with RES, PUA NPs and PUA NPs@RES significantly inhibited the green fluorescence intensity and enhanced the red fluorescence (Fig. 3D), indicating that these treatments could inhibit apoptosis by restoring mitochondrial function. The flow cytometry results of JC-1 were also consistent with the fluorescence results (Fig. 3E and Additional file 1: S8). Furthermore, we evaluated the protective effect of each group on oxidative stress-induced apoptosis using flow cytometry. As shown in Fig. 3F, the H_2_O_2_ group exhibited the highest apoptosis level, indicating that excessive ROS had a significant effect on cell apoptosis. However, after treatment with RES, PUA NPs and PUA NPs@RES, apoptosis was significantly reduced. The quantitative statistical analysis showed that the proportion of HK‐2 cell apoptosis was significantly reduced in each group, especially in the PUA NPs@RES group, confirming that all groups play a protective role by reducing intracellular ROS level (Additional file 1: Fig. S9).

### In vivo biodistribution of PUA NPs

To investigate the in vivo biodistribution of PUA NPs, we first established AKI mouse model by injecting 50% glycerol into the hind limb of BALB/c mice, which induced rhabdomyolysis, causing oxidative stress and eventually AKI [29]. Fluorescence probe DiR was encapsulated into PUA NPs and formed DiR-NPs. Normal and AKI mice were intravenously injected with free DiR and DiR-NPs and visualized using the in vivo imaging system.

**Fig. 4:**
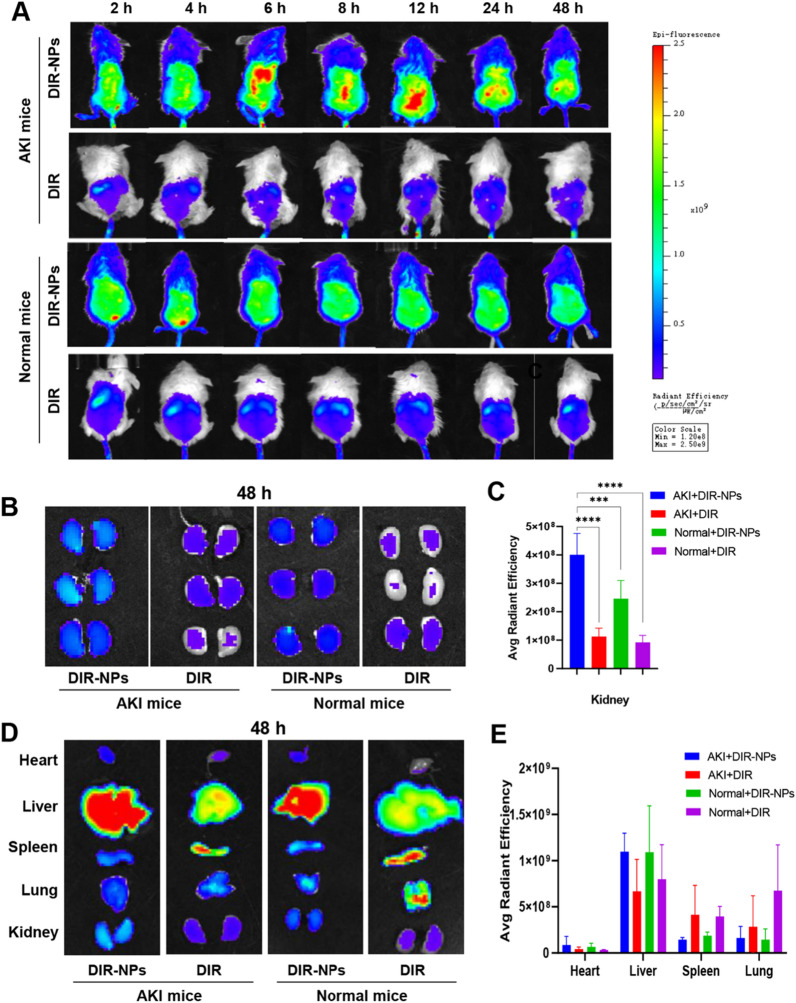
In vivo biodistribution of PUA NPs. **A** Fluorescence images of AKI and normal mice following the injection of DiR-NPs and free DiR at different time points. **B** Fluorescence images of the kidney tissues dissected from AKI and normal mice at 48 h. **C** Quantitative analysis of fluorescence intensity of kidney tissues at 48 h. **D** Fluorescence images of the major organs dissected from AKI and normal mice at 48 h. **E** Quantitative analysis of fluorescence intensity in major organs at 48 h

Figure 4A shows the real-time distribution of free DiR and DiR-NPs in the normal and AKI mice, with the most prominent accumulation observed in the liver. After 48 h, kidneys and other major organs were collected and imaged. As shown in Fig. 4B and C, the accumulation of DiR-NPs in the AKI group was approximately 1.6 times higher than that in the normal group, while the free DiR fluorescence signal in AKI group did not significantly differ from that in the normal group. This is primarily attributed to increased vascular permeability induced by kidney injury, facilitating the enhanced accumulation of PUA NPs. In Fig. 4D and E, the fluorescence intensity of DiR and DiR-NPs in major organs is presented. It is worth noting that even after 48 h, both DiR and DiR-NPs exhibited significant fluorescence levels in the livers. This can be attributed to two main factors: firstly, the complete metabolism of DiR in vivo often requires an extended period; secondly, the liver is a major component of the reticuloendothelial system and houses a significant population of Kupffer cells, which can efficiently capture the foreign substances from the bloodstream [30–32]. These factors contribute to the noticeable retention in the liver over time. Despite encountering clearance by Kupffer cells, a substantial quantity of DiR-NPs managed to overcome physiological barriers and accumulated in the kidneys of AKI mice for potential therapeutic action.

### In vivo therapeutic efficacy of PUA NPs@RES

**Fig. 5:**
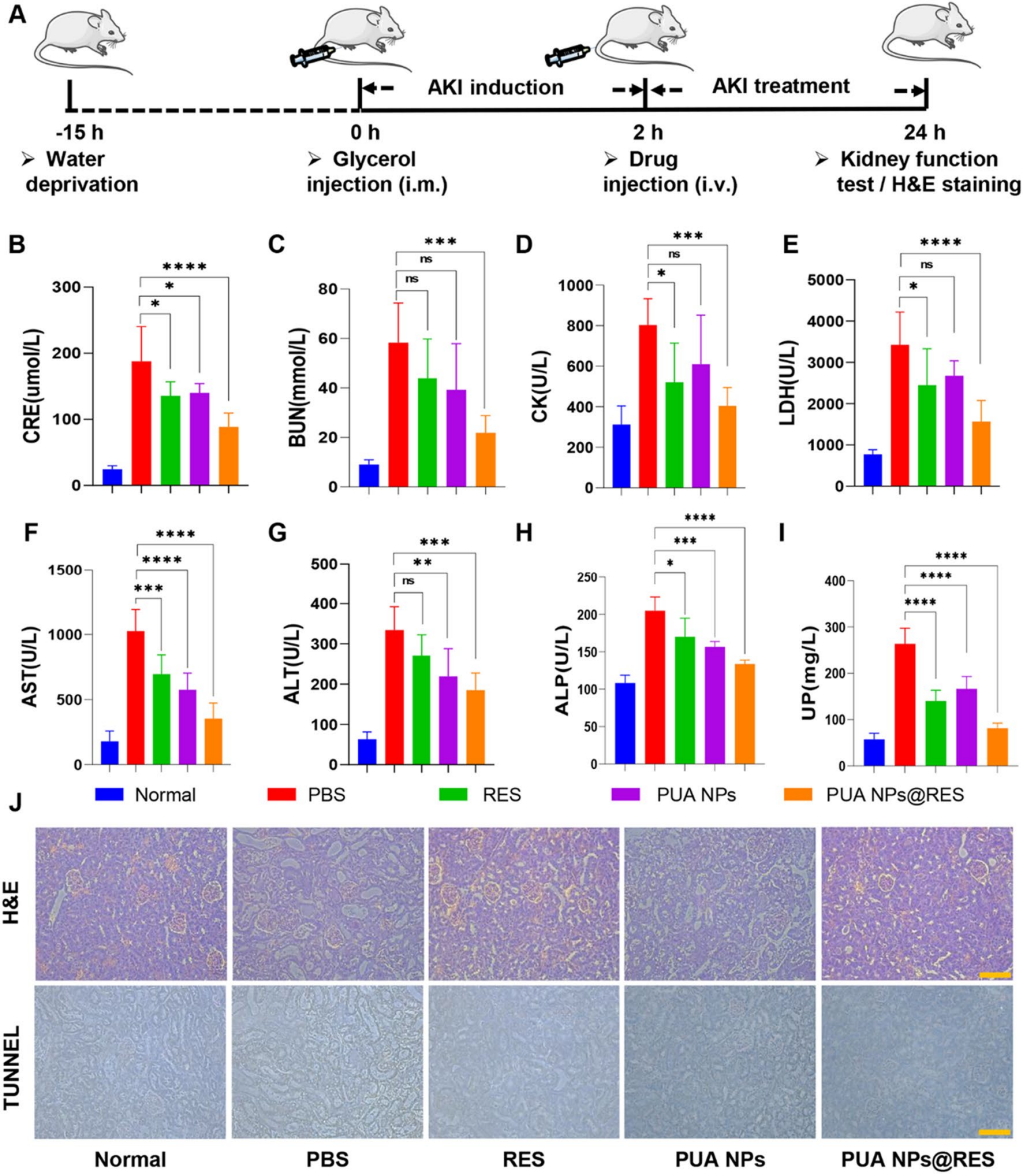
Histopathological assessment on kidney function and anti-apoptotic efficacy. **A** Schematic illustration depicting the establishment of AKI mouse models and the subsequent therapeutic regimen. **B** CRE, **C** BUN, **D** CK, **E** LDH, **F** AST, **G** ALT, **H** ALP, **I** UP levels in AKI mice at 24 h after treatment with RES, PUA NPs and PUA NPs@RES (n = 5). **J** Representative histological **H and E** and apoptosis (TUNEL) staining images of renal tissues collected from different treatment groups. Scale bar: 100 μm

The in vivo therapeutic efficacy of PUA NPs@RES against AKI was assessed by blood biochemical and histological analysis. Following AKI modeling, four groups were randomly assigned and intravenously injected with PBS, PUA NPs, free RES and PUA NPs@RES. Blood, urine and kidney samples were collected 24 h later for further evaluation (Fig. 5A). As shown in Fig. 5B–I, compared with normal mice, the development of AKI significantly elevated the blood and urine biochemical markers of mice, including creatinine (CRE), blood urea nitrogen (BUN), creatine kinase (CK), lactate dehydrogenase (LDH), alanine amiotransferase (AST), alanine aminotransferase (ALT), alkaline phosphatase (ALP), and urinary protein (UP). However, after treatments with RES, PUA NPs, and PUA NPs@RES, these parameters greatly declined, suggesting effective relief of AKI progression. Consistent with the in vitro results, PUA NPs@RES was found to be more effective in reducing CRE, BUN, CK, LDH and UP levels, compared with free RES. Moreover, PUA NPs also exhibited a certain degree of reduction in CRE, BUN, CK, LDH and UP levels, possibly attributed to PUA's ability to scavenge ROS. In addition, similar to the improvements observed in renal function, biochemical measurements of liver function confirmed that free RES, PUA NPs, and PUA NPs@RES were all able to ameliorate oxidative stress-induced liver injury.

Hematoxylin and eosin (H&E) and terminal deoxynucleotidyl transferase dUTP nick-end labelin (TUNEL) staining were further conducted to evaluate therapeutic efficacy (Fig. 5J). Among all treatment groups, especially in the PUA NPs@RES treatment group, the degree of injury was significantly reduced, and the structures of glomeruli and proximal tubules were well preserved. Apoptosis of kidney tissue is a common pathological phenomenon in AKI [33]. As evidenced by the TUNEL staining results, a large number of positive cells were observed in the AKI group, while each treatment group showed varying degrees of improvement., Notably, PUA NPs@RES treatment group exhibited the least apoptotic cell content compared to the other two groups. Taken together, these results demonstrated the great potential of PUA NPs@RES in treating AKI.

### Therapeutic mechanisms of PUA NPs on AKI

**Fig. 6:**
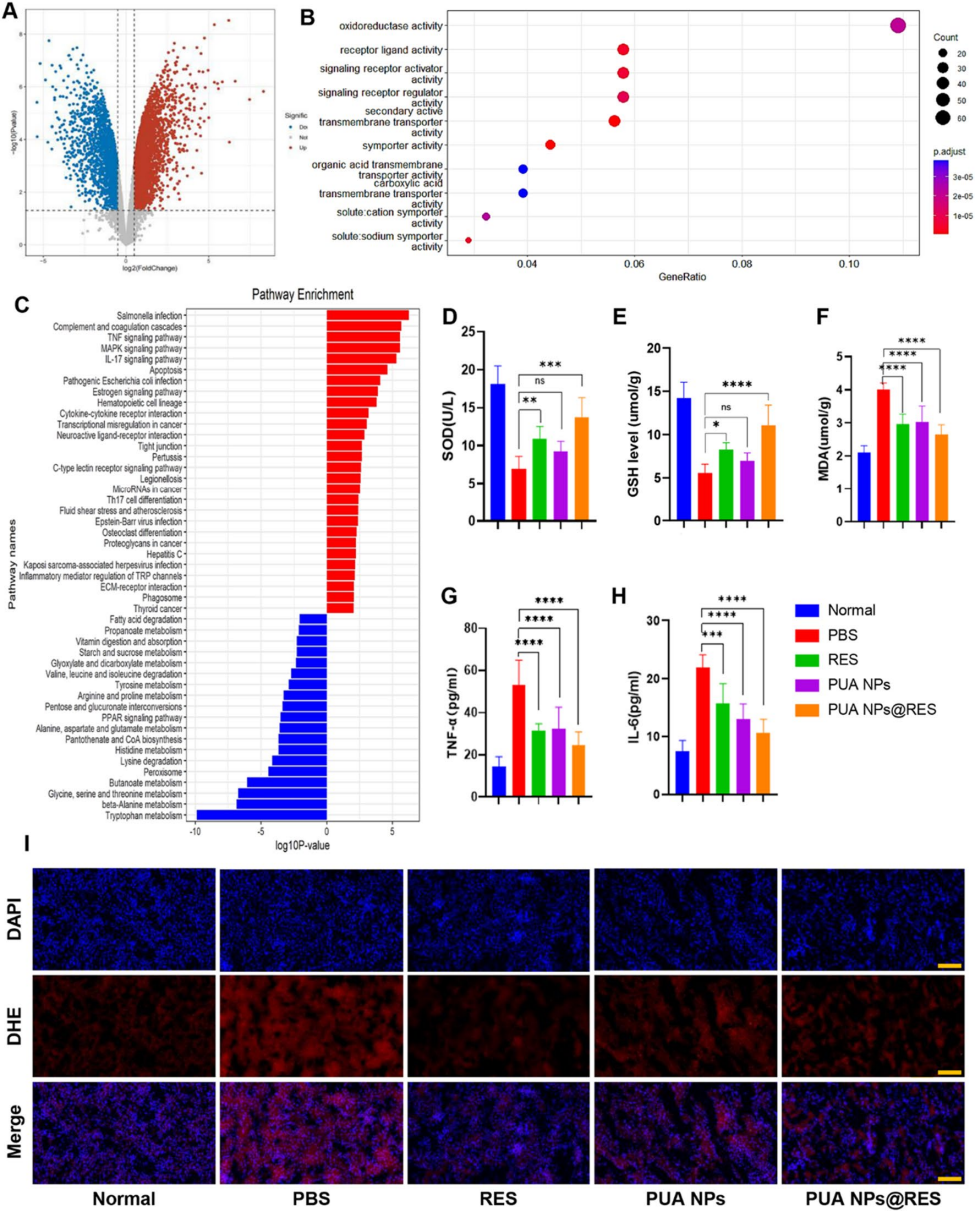
Therapeutic mechanisms of PUA NPs on AKI. **A** Volcano plots showing the identified upregulated and downregulated genes by PUA NPs. **B** Gene ontology analysis of molecular functions of PUA NPs. **C** KEGG pathway enrichment analysis of the identified differentially expressed genes. The most significantly enriched pathways are shown. The levels of **D** SOD, **E** GSH, **F** MDA, **G** TNF-α, **H** IL-6 in the kidney after different treatments. **I** ROS levels in kidney tissues from different treatments groups stained by DHE. Scale bar: 100 µm

To further investigate potential therapeutic mechanisms against AKI, transcriptomic analysis was performed comparing the AKI control group and the PUA NPs treatment group. The volcano map (Fig. 6A) showed significant differences in the expression of 704 genes, with 331 genes down-regulated and 373 genes up-regulated. Subsequently, we conducted a cell biology function enrichment analysis through Gene Ontology (GO) database (Fig. 6B), suggesting that oxidoreductase activity is highly correlated with the therapeutic mechanism of PUA NPs. Therefore, SOD, GSH and MDA levels in kidney tissue were detected to reflect oxidoreductase activity in the body. The activities of SOD and GSH in kidney tissue of AKI mice in the positive control group exhibited a significant decrease. However, in all treatment groups, including RES, PUA NPs, and PUA NPs@RES, the activities of SOD and GSH in the kidney were increased. Particularly, the PUA NPs@RES treatment group exhibited the highest levels of SOD and GSH activities (Fig. 6D and E). Conversely, MDA levels increased in AKI mice of the positive control group, while decreased in all treatment groups, especially in the PUA NPs@RES group, further demonstrating that RES, PUA NPs, and PUA NPs@RES can protect cells from lipid peroxidation damage by effectively scavenging ROS (Fig. 6F).

Dihydroethidine (DHE) staining was carried out to visualize ROS levels in kidney sections. Compared to the PBS group, ROS levels in renal tissue of all treatment groups were decreased to varying degrees. Notably, the PUA NPs@RES treatment group exhibited the most significantly inhibition of ROS levels (Fig. 6I). These findings suggest that RES, PUA NPs, and PUA NPs@RES can protect kidney cells of AKI mice by eliminating ROS and maintaining oxidoreductase activity in vivo.

Heat map (Additional file 1: Fig. S10) and Kyoto Encyclopedia of Genes and Genomes (KEGG) pathway enrichment analysis (Fig. 6C) revealed a strong correlation between the therapeutic mechanism of PUA NPs and the MAPK and TNF signaling pathways. Gene Set Enrichment Analysis (GSEA) (Additional file 1: Fig. S11) further revealed that PUA NPs group had more genes associated with MAPK signaling pathways and stronger associations than the control group. Reportedly, ROS can activate MAPK signaling pathway to induce apoptosis of kidney cells and release local or systemic inflammatory mediators to aggravate kidney injury [34, 35]. In addition, ROS have been reported to promote the production of pro-inflammatory cytokines such as TNF-α and IL-6 [36, 37]. Subsequently, TNF-α can further trigger a robust inflammatory cascade through the TNF-α/MAPK signaling pathway, leading to an excessive inflammatory response and more severe kidney injury [38]. Therefore, by detecting the levels of pro-inflammatory cytokines TNF-a and IL-6 in mouse kidney tissue, we found that RES, PUA NPs and especially PUA NPs@RES can effectively reduce the levels of inflammatory cytokines in kidney tissue, thus alleviating kidney injury (Fig. 6G and H).

### In vivo biosafety assessment

**Fig. 7:**
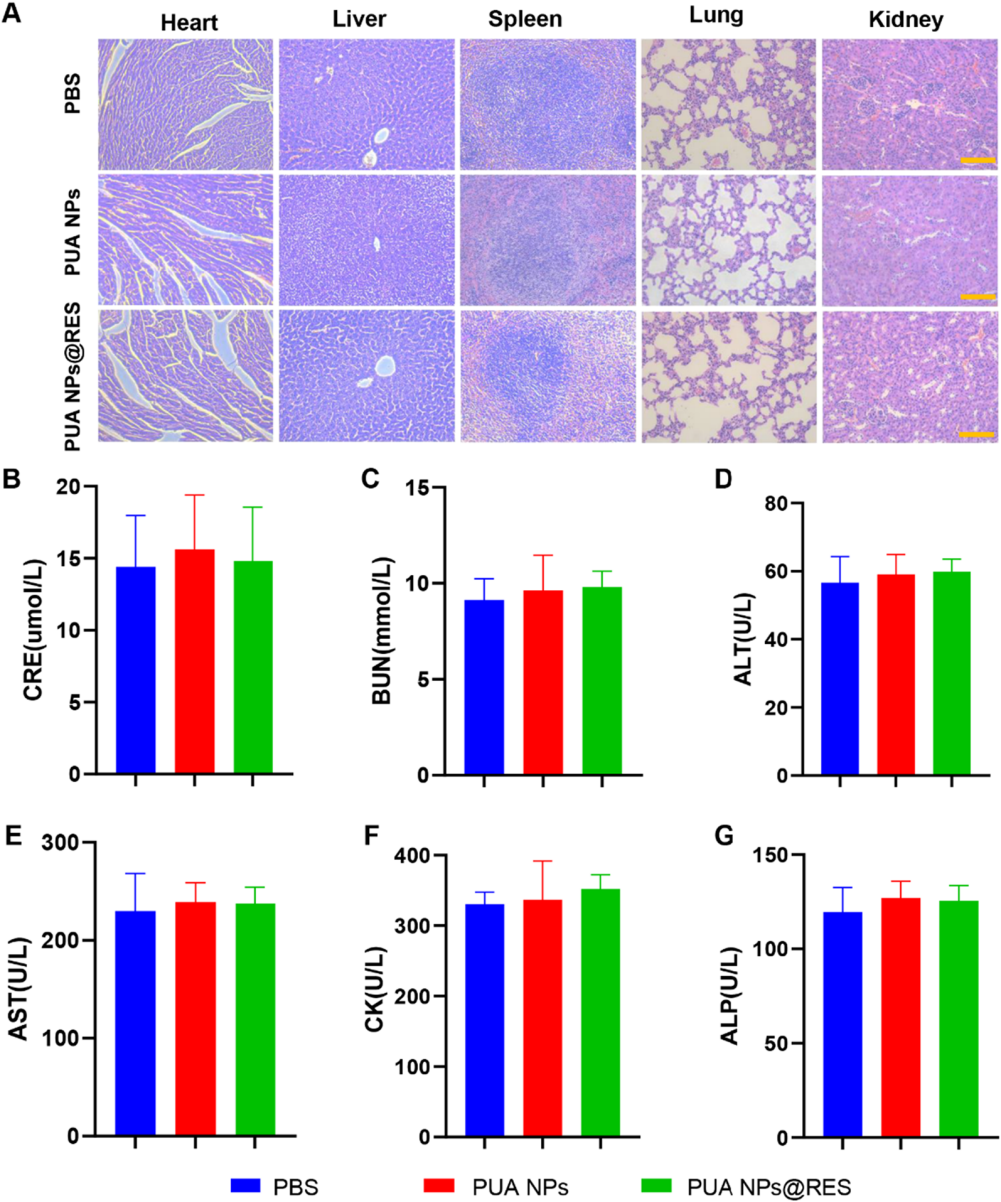
In vivo toxicity assessment of PUA NPs and PUA NPs@RES. **A** Histopathology images of heart, liver, spleen, lung, and kidney collected from different treatment groups, scale bar = 100 μm. Serum levels of **B** CRE, **C** BUN, **D** ALT, **E** AST, **F** CK, **G** ALP in normal mice from different treatment groups

In order to evaluate the long-term biosafety of PUA NPs and PUA NPs@RES in vivo, the major organs (heart, liver, spleen, lung and kidney) of mice were collected for H&E staining after high-dose administration for 21 days. As shown in Fig. 7A, compared with the control group, neither the PUA NPs nor PUA NPs@RES groups exhibited significant tissue damage, indicating that no visible organ lesions occurred after long-term injection. To further quantitatively confirm the safety of PUA NPs and PUA NPs@RES, we collected the serum from mice and detected the blood biochemical indexes (CRE, BUN, ALT, AST, CK, ALP). The results showed that liver and kidney function indexes of mice in the three groups remained within the normal range (Fig. 7B–G). Additionally, there were no significant differences in body weight between the three groups (Additional file 1: Fig. S12). Overall, these findings collectively demonstrate the good biocompatibility and safety of both PUA NPs and PUA NPs@RES in vivo.

## Conclusion

In conclusion, we successfully synthesized PUA polymer from naturally derived UA compound with anti-inflammatory activity, and systematically investigated the potential of PUA NPs as therapeutic nanocarriers to improve drug delivery efficiency and achieve synergistic therapeutic effects in AKI therapy. PUA NPs can effectively load hydrophobic antioxidant and anti-inflammatory drugs (such as RES), resulting in favorable stability and exhibiting good antioxidant, anti-inflammatory effects in vitro. Moreover, PUA NPs can effectively improve the accumulation of RES in the kidney, exert inherent antioxidant properties, and further enhance the therapeutic effect of RES in AKI treatment. The mechanism may be related to the improvement of oxidoreductase activity and the inhibition of MAPK and TNF signaling pathways. The findings of this study will facilitate the development of therapeutic nanocarriers containing antioxidant drugs as promising treatments for AKI.

### Supplementary Information

**Additional file 1: Table S1**. Molecular weight information of PUA polymer. **Table S2**. The influence of drug/carrier ratio on DLC and DLE of PUA NPs@RES. **Fig. S1** 1H-NMR spectra of UA in DMSO-d6. **Fig. S2** 1H-NMR spectra of PUA polymer in DMSO-d6. **Fig. S3** FT-IR spectra of PUA and UA monomer. **Fig. S4** TEM image of PUA NPs. **Fig. S5** Cell viability of HK-2 cells treated free RES, PUA NPs and PUA NPs@RES across various equivalent RES concentrations for 24 h. **Fig. S6** Quantitative analysis of cellular uptake of H2O2-stimulated HK-2 cells detected by flow cytometry. **Fig. S7** Quantitative analysis of intracellular ROS levels of H2O2-stimulated HK-2 cells detected by flow cytometry. **Fig. S8** Quantitative analysis of mitochondrial membrane potential of H2O2-stimulated HK-2 cells after different treatments. **Fig. S9** Florescence analysis of Cell apoptosis after different treatments. **Fig.S10** Heat map results of upregulated and downregulated genes after PUA NPs treatment (fold change ≥2 and P < 0.05). **Fig. S11** GSEA enrichment plots of gene set involved in MAPK signaling pathway. **Fig. S12** Changes in body weight of mice following with PBS, PUA NPs and PUA NPs@RES for 21 days.

## Data Availability

All data generated or analyzed during this study are included in this published article and its additional information files.
