# Correction: Natural ursolic acid based self-therapeutic polymer as nanocarrier to deliver natural resveratrol for natural therapy of acute kidney injury

**DOI:** 10.1186/s12951-024-02321-x

**Published:** 2024-03-01

**Authors:** Yuanpeng Nie, Liying Wang, Shengbo Liu, Chunlei Dai, Tianjiao Cui, Yan Lei, Xinru You, Xiaohua Wang, Jun Wu, Zhihua Zheng

**Affiliations:** 1 Department of Nephrology, Center of Kidney and Urology, The Seventh Affiliated Hospital, Sun Yat-Sen University, Shenzhen, 518107 China; 2 Department of Hematology, The Seventh Affiliated Hospital, Sun Yat-Sen University, Shenzhen, 518107 China; 3 Department of Thoracic Surgery, Guangdong Provincial People’s Hospital (Guangdong Academy of Medical Sciences), Southern Medical University, Guangzhou, 510080 China; 4 School of Biomedical Engineering, Sun Yat-Sen University, Shenzhen, 518107 China; 5 Center for Nanomedicine and Department of Anesthesiology, Brigham and Women’s Hospital, Harvard Medical School, Boston, MA 02115 USA; 6 Bioscience and Biomedical Engineering Thrust, The Hong Kong University of Science and Technology (Guangzhou), Nansha, Guangzhou 511400 China; 7 Division of Life Science, The Hong Kong University of Science and Technology, Hong Kong SAR,, China

**Correction: Journal of Nanobiotechnology (2023) 21:484**

10.1186/s12951-023-02254-x

In this article Yuanpeng Nie and Liying Wang should have been denoted as equally contributing authors.

The original article [1] has been revised.
