# Supplementary material for: Natural ursolic acid based self-therapeutic polymer as nanocarrier to deliver natural resveratrol for natural therapy of acute kidney injury
Source: J Nanobiotechnology. 2023 Dec 17;21:484. doi: 10.1186/s12951-023-02254-x (PMC10726514; doi:10.1186/s12951-023-02254-x)
Supplement: Supplementary file 1 — Additional file 1: Table S1. Molecular weight information of PUA polymer. Table S2. The influence of drug/carrier ratio on DLC and DLE of PUA NPs@RES. Fig. S1 1H-NMR spectra of UA in DMSO-d6. Fig. S2 1H-NMR spectra of PUA polymer in DMSO-d6. Fig. S3 FT-IR spectra of PUA and UA monomer. Fig. S4 TEM image of PUA NPs. Fig. S5 Cell viability of HK-2 cells treated free RES, PUA NPs and PUA NPs@RES across various equivalent RES concentrations for 24 h. Fig. S6 Quantitative analysis of cellular uptake of H2O2-stimulated HK-2 cells detected by flow cytometry. Fig. S7 Quantitative analysis of intracellular ROS levels of H2O2-stimulated HK-2 cells detected by flow cytometry. Fig. S8 Quantitative analysis of mitochondrial membrane potential of H2O2-stimulated HK-2 cells after different treatments. Fig. S9 Florescence analysis of Cell apoptosis after different treatments. Fig.S10 Heat map results of upregulated and downregulated genes after PUA NPs treatment (fold change ≥2 and P < 0.05). Fig. S11 GSEA enrichment plots of gene set involved in MAPK signaling pathway. Fig. S12 Changes in body weight of mice following with PBS, PUA NPs and PUA NPs@RES for 21 days. [file 12951_2023_2254_MOESM1_ESM.docx]

**Natural ursolic acid based self-therapeutic polymer as nanocarrier to deliver natural resveratrol for natural therapy of acute kidney injury**

Yuanpeng Nie^# a^, Liying Wang^# b^, Shengbo Liu^c^, Chunlei Dai^d^, Tianjiao Cui^a^, Yan Lei^a^, Xinru You ^e^, Xiaohua Wang^a^, Jun Wu * ^b,f,g^, and Zhihua Zheng* ^a^

**Methods**

**Synthesis of Poly(Ursolic Acid) Polymer:** Poly(ursolic acid) polymer was prepared via the polycondensation of ursolic acid under the catalysis of thionyl chloride and anhydrous pyridine as an acid binding agent. The synthetic route was as shown in Figure 1a: in a well-ventilated environment, anhydrous pyridine (10 mL) was added to a round-bottom flask (50 mL) and stirred in an ice bath for 5 min before the addition of thionyl chloride (0.33 g, 2.76 mmol). After an additional 15 min of stirring in ice bath, white powder of UA (0.42 g, 0.92 mmol) was added and stirred until dissolved well. After stirring at room temperature for 2 h, the reaction solution was transferred to a 500 mL beaker and washed with 1 m HCl and deionized water (six times). After suction filtration and lyophilization, the product PUA was obtained as a pale yellow solid (0.38 g, yield: 90%).

The PUA structure was confirmed by ^1^H-nuclear magnetic resonance(1H-NMR; Bruker Avance Ⅲ 400, Germany), and Fourier transform infrared spectroscopy (FTIR, Bruker, Germany). The weight-average molecular weights and PDI of PUA were measured by gel permeation chromatography (GPC; Agilent, USA).

**Preparation and characterization of NPs**: Blank NPs (PUA NPs) and PUA NPs @RES were both prepared by the nanoprecipitation method. Briefly, PUA polymer, RES, and DSPE-PEG 2k were separately dissolved in DMSO at the same concentration of 10 mg/mL and mixed in a specific volume ratio. Then 640 μL of the mixture was dropwise added into 12.8 mL of ultra-pure water under constant stirring. The NPs solution was ultra-filtered twice using an ultrafiltration centrifugal tube (Millipore, MWCO 10k Da). After removing DMSO and the free molecules, the concentrated NPs solution was suspended in PBS for further characterization and use. Transmission electron microscopy (TEM; JEOL Ltd, Japan) was applied to visualize the morphology of NPs and dynamic light scattering (DLS; Zetasizer Nano-ZS90, Malvern, UK) was used to measure the particle size. To evaluate the stability of NPs, PUA NPs and PUA NPs @RES dispersed in PBS and PBS containing 10% FBS were measured by DLS at prearranged time intervals for seven days.

**Drug loading capacity of PUA NPs@RES**: The drug loading capacity of PUA NPs@RES was determined by HPLC. Briefly, DTX@PCA NPs were dissolved in methanol and the NP structure was completely broken by ultrasound to release the encapsulated RES.Next, the sample solution was centrifuged at 12000 rpm for 10 min. The RES content was detected under a mobile phase of water/ methanol=35/65 and UV detection wavelength of 230 nm. The drug loading capacity (DLC) and drug encapsulation efficiency (DLE) were calculated using the following formulae:

DLC(wt%)=amount of encapsulated drug/amount of nanoparticles × 100%

DLE(wt%)=amount of encapsulated drug/amount of feeding drug × 100%

**Drug release behavior of PUA NPs@RES:** The in vitro drug release behavior of PUA NPs @RES was investigated using the dialysis method. Briefly, 1 mL of PUA NPs @RES solution was transferred into a dialysis bag (MWCO 3500 Da) and then immersed in PBS containing 0.1% Tween 80 with different pH values (4.5, 5.0, and 7.4). The drug release process was performed in a thermostatic shaker with a temperature of 37 ℃ and a constant shaking speed of 100 rpm. At prearranged time intervals, 1mL of sample solution was withdrawn, followed by the supplement of the corresponding release medium. Finally, the released RES was determined by HPLC and expressed as the percentage of the cumulative amount.

**Hemolysis test:** A hemolysis test was performed to evaluate the hematotoxicity of PUA NPs as nanocarriers. Briefly, fresh whole blood obtained from SD rats was centrifuged at 1,000 rpm for 5 min. Next, the plasma and buffy coat layer were discarded, and the remaining red blood cells (RBCs) were further washed using PBS till the supernatant became clear. Finally, the concentrated RBCs were diluted to 2% in PBS. Thereafter, 1 mL of PUA NPs dispersed in PBS, with concentrations ranging from 31.25 to 1000 μg/mL, was mixed with an equal volume of RBC suspension. The RBC suspension mixed with pure PBS (0% hemolysis) and ultrapure water (100% hemolysis) was set as negative control and positive control, respectively. All the samples were placed in a constant temperature oscillator with a temperature of 37 °C and a constant shanking speed of 100 rpm. After incubation for 3 h, the mixture was centrifuged at 1,000 rpm for 5 min to collect the supernatant. Finally, the absorbance of each group was measured at 541 nm using a microplate reader. The hemolysis percentage was calculated as follows:

$$Hemolysis[\%]=\frac{{(Ab}_{\mathrm{sample}}-\mathrm{Ab}_{negative control})}{{(Ab}_{positive control}-\mathrm{Ab}_{negative control}）}\times100\%$$

**Cell lines and culture conditions：**Human Kidney-2(HK-2) cells were obtained from American Type Culture Collection (ATCC), barring special circumstances, incubated in DMEM containing 10%FBS and 1% P/S and maintained at 37℃, under 5% CO2.The HK-2 cells were cultured in Dulbecco’s modified Eale’s medium (DMEM) supplemented with 10% fetal bovine serum (FBS), 1% streptomycin, and penicillin at 37 °C in an incubator under 5% CO_2_.

**Cytotoxicity of PUA NPs @RES in Vitro：**Human renal tubular epithelial cell line HK2 cells were seeded in 96-well plates at 5 × 103 cells per well and cultured with Dulbecco’s modified Eagle’s medium containing 10% fetal bovine serum and 1% penicillin–streptomycin and incubated at 37 °C under 5% CO_2_ in a water-saturated atmosphere for 24 h. Cells were cultured for 1 day and then treated with RES、PUA NPs or PUA NPs @RES of various concentrations at 37 °C under 5% CO_2_ for 24 h. Cell viability was evaluated by the CCK8 assay.

**DPPH free radical scavenging test：**Simply put, DPPH is dissolved in anhydrous ethanol and diluted into 80ug/ml. Experimental group At: take the mixture of 1ml DPPH solution and 1ml material, shake for 30 minutes; blank group: 1ml anhydrous ethanol and 1ml material mixture, shake for 30 minutes; background group Ab: take 1ml DPPH solution and 1ml ultra-pure water, shake for 30 minutes; 200ul solution was applied to 96-well plate, and the wavelength of enzyme meter was set at 517nm. and determine the optical absorption value of each well.

Calculation formula:

$$Clearance rate[\%]=(1-\frac{At-Ac}{\mathrm{Ab}})\times100\%$$

**ABTS Radical Cation Decolorization Assay：**ABTS at 7 mM concentration was dissolved in water before 2.45 mM potassium persulfate (final concentration) was added to produce the ABTS radical cation (ABTS•+). The stock solution was kept in the dark at room temperature for 12 h before use and then the ABTS•+ solution was diluted with PBS to achieve a final absorbance value of ∼0.75 at 734 nm with a volume of 200 μL in each well of a standard 96-well plate. Different concentrations of RES, PUA NPs and PUA NPs@RES were added into ABTS•+ solution.Record the absorbance of each hole at 734nm. A: the absorbance of sample solution of each concentration. A0: absorbance of solution in blank control.

Calculation formula:

$$Clearance rate[\%]=\frac{(A_{0}-A)}{A_{0}}\times100\%$$

**Cellular uptake study：**Coumarin 6 (C6) was used as a fluorescence probe to label the PUA NPs and track the cellular internalization behavior of NPs on HK2 cells. HK2 cells with a density of 1×105 cells/dish were inoculated on a 6-well plate and incubated overnight at 37 °C. Then the culture medium was removed and replaced by serum-free medium containing C6-loaded PUA NPs (C6@PUA NPs, at a C6 dose of 0.2 μg/mL). After incubation for 1, 4, and 8 h, the cells were washed twice with pre-cooling PBS and Finally, the cells were maintained in PBS, followed by the imaging of fluorescent microscope (Leica, Germany). The ROS levels in HK2 cells were quantitatively determined by flow cytometry.

**Study on ROS scavenging ability in vitro：**2′,7′-Dichlorofluorescin diacetate (DCFH-DA), an oxidation sensitive fluorescent dye, was used to detect the intracellular ROS level. Briefly, DCFH-DA is a non-fluorescent chemical compound which could diffuse through cell membrane freely and could be hydrolyzed by intracellular esterase to DCFH. The non-fluorescent DCFH could be oxidized by the intracellular ROS to fluorescent DCF. Therefore, the quantity of intracellular ROS is correlated with the fluorescent intensity of DCF. After the aforementioned incubation with H_2_O_2_ for 24 h, cells were gently rinsed thrice with serum-free medium to remove the free RES, PUA NPs and PUA NPs@RES. Then, a final concentration of 10 μM of DCFH-DA in serum-free medium was added to the cells and incubated in dark at 37 °C for 30 min. Afterwards, the cells were washed with serum-free medium thrice to remove unloaded DCFH-DA probe, then were imaged using a fluorescent microscope (Leica, Germany), and were subjected to a flow cytometry analysis to quantify the intracellular ROS levels respectively.

**Detection of pro-inflammatory cytokines and oxidative stress：**The levels of tumor necrosis factor-α (TNF-α), interleukin-6 (IL-6) as pro-inflammatory cytokines were measured using ELISA kits according to the protocol provided by the manufacturer (NOVUS, USA). The levels of SOD, MDA and GSH were measured using commercial kits according to the manufacturer's protocol (Beyotime Biotechnology, China). All samples were measured triplicate.

**Antiapoptotic activity in vitro**

**Mitochondrial Membrane Potential (Δψm) Measurement:** HK2 were pretreated with H_2_O_2_ (250 μM) for 2h, and then cultured for 24h with different treatments. Mitochondrial membrane potential was determined using the protocol and reagent provided by the JC-1 kit (Beyotime Biotech, China). The cells were observed with a fluorescence microscope. Finally,the JC-1 levels in HK2 cells were quantitatively determined by flow cytometry.

**Apoptosis:** HK2 were pretreated with H_2_O_2_ (250 μM) for 2 h and incubated with different treatments for 24 h. Annexin V-FITC/PI Apoptosis detection kit (Beyotime Biotech, China) was used to detect the apoptosis effect of PUA NPs@RES. Finally, the cells were quantitatively detected by flow cytometry.

**Animal and rhabdomyolysis (RM)-induced AKI model**

The Animal Ethics Committee of the Laboratory Animal Center of Sun Yat-sen University approved the protocols of all animal studies conducted in this study. All animal experiments were conducted using female BALB/c mice, which were purchased from the Laboratory Animal Center of Sun Yat-sen University.

**RM-induced AKI model:：**Mice did not drink water for 15 h, but were given food. After that, 50% glycerol (8 mL/kg) was injected into the mouse hindlimb. After 24 hours, the mice were euthanized and their major organs and blood were collected.

**Study on biological distribution in vivo:** Study on biological distribution in vivo of AKI mice and normal mice, using DiR as fluorescent probe to simulate free drugs, and tracking the biological distribution of PUA NPs in vivo. Simply, the mice were randomly divided into four groups (n=3), two groups were normal and two groups were AKI mice, which were injected intravenously with free DiR solution (0.4mg/kg) and DIR-loaded PUA NPs (DiR@PUA NPs) at the same dose of DiR. At predetermined intervals (2, 4, 6, 8, 12, 24, and 48 h), the mice were anesthetized and photographed using an in vivo imaging system (IVIS, PerkinElmer, USA) at the excitation/emission (Ex/Em) wavelength of 740/790nm. The mice were killed 48 hours after injection. Kidneys and major organs (heart, liver, spleen and lungs) were collected and photographed at the same Ex/Em wavelength.

**Therapeutic effect of PUA NPs@RES on AKI mice**

**Evaluation of kidney function:** The mice were divided into 5 groups (n = 5): The first group was normal control group: healthy mice were injected with PBS; the second group was RM-AKI mice injected with PBS.The third to fifth groups were RM-AKI mice injected with RES, PUA NPs and PUA NPs@RES, respectively. Mice did not drink water for 15 h, but were given food. After that, 50% glycerol (8 mL/kg) was injected into the mouse hindlimb. All mice were given intravenous medication 2 hours after glycerol injection. The content of RES in the RES group and the PUA NPs@RES group was the same, both of which were 5mg/kg. Ultimately, the serum and urine of mice were collected to detect biochemical parameters, such as urea nitrogen (BUN), creatinine (CRE), alanine aminotransferase (ALT), aspartate transaminase (AST), creatine kinase (CK), alkaline phosphatase (ALP) and urinary protein (UP). The major organs were fixed in 4% paraformaldehyde for hematoxylin and eosin (H&E) staining and terminal deoxynucleotidyl transferase biotin-dUTP nick end labeling (TUNEL) assay. In addition, the in vivo toxicity tests were conducted via normal tissue sections and serum biochemistry analysis. After the in vivo therapeutic test, the mice were sacrificed, and blood and major organs were collected.

**Renal tissues analysis after treatment:** The mouse kidneys were frozen and stored in a refrigerator at-80 °C. Renal tissue homogenate is produced according to the requirements of different detection schemes. The levels of tumor necrosis factor-α (TNF-α), interleukin-6 (IL-6) as pro-inflammatory cytokines were measured using ELISA kits according to the protocol provided by the manufacturer (NOVUS, USA).The levels of SOD, MDA and GSH were measured using commercial kits according to the manufacturer's protocol (Beyotime Biotechnology, China).To detect the production of superoxide free radicals in the body. The renal tissue was prepared into frozen sections with a thickness of 5 μm and incubated with 0.3% Triton Xmuri 100 at room temperature for 15 min. After the solution was removed, the slices were cut with Dihydroethidium (Beyotime Biotechnology, Shanghai) for 30 minutes. After washing PBS for three times, the tissue was incubated with DAPI (Beyotime Biotechnology, Shanghai), and the fluorescence was observed under fluorescence microscope.

**Transcriptome analysis of AKI mice:** BALB/c mice with AKI model were randomly divided into PBS injection group (control group, n =3) and PUA NPs injection group (experimental group, n =3). The mice were sacrificed 24 h after injection and the kidneys were collected. Kidney RNA purification, reverse transcription, library construction and sequencing were all conducted in BGI (Shenzhen, China).

**Statistical analysis:** The statistical analysis of data was conducted using SPSS software. Two groups were compared using the two-tailed Student’s t-test. One-way ANOVA was applied to analyze multiple groups. Significant difference was displayed as **p* ≤ 0.05, ***p* < 0.01, *** *p* < 0.001 or **** *p* < 0.0001.

Table S1. Molecular weight information of PUA polymer

| Polymer | Mn | Mw | Mz | PDI |
| --- | --- | --- | --- | --- |
| PUA | 2453 | 2457 | 2461 | 1.00 |

Table S2. The influence of drug/carrier ratio on DLC and DLE of PUA NPs@RES

| Ratio | 1：3 | 1：5 | 1：7 | 1：9 |
| --- | --- | --- | --- | --- |
| DLC (%) | 3.84±0.04 | 2.94±0.02 | 1.95±0.03 | 1.91±0.06 |
| DLE (%) | 15.37±0.18 | 17.71±0.17 | 15.63±0.2 | 19.06±0.60 |

DLC: drug-loading capacity.

DLE: drug-loading efficiency.


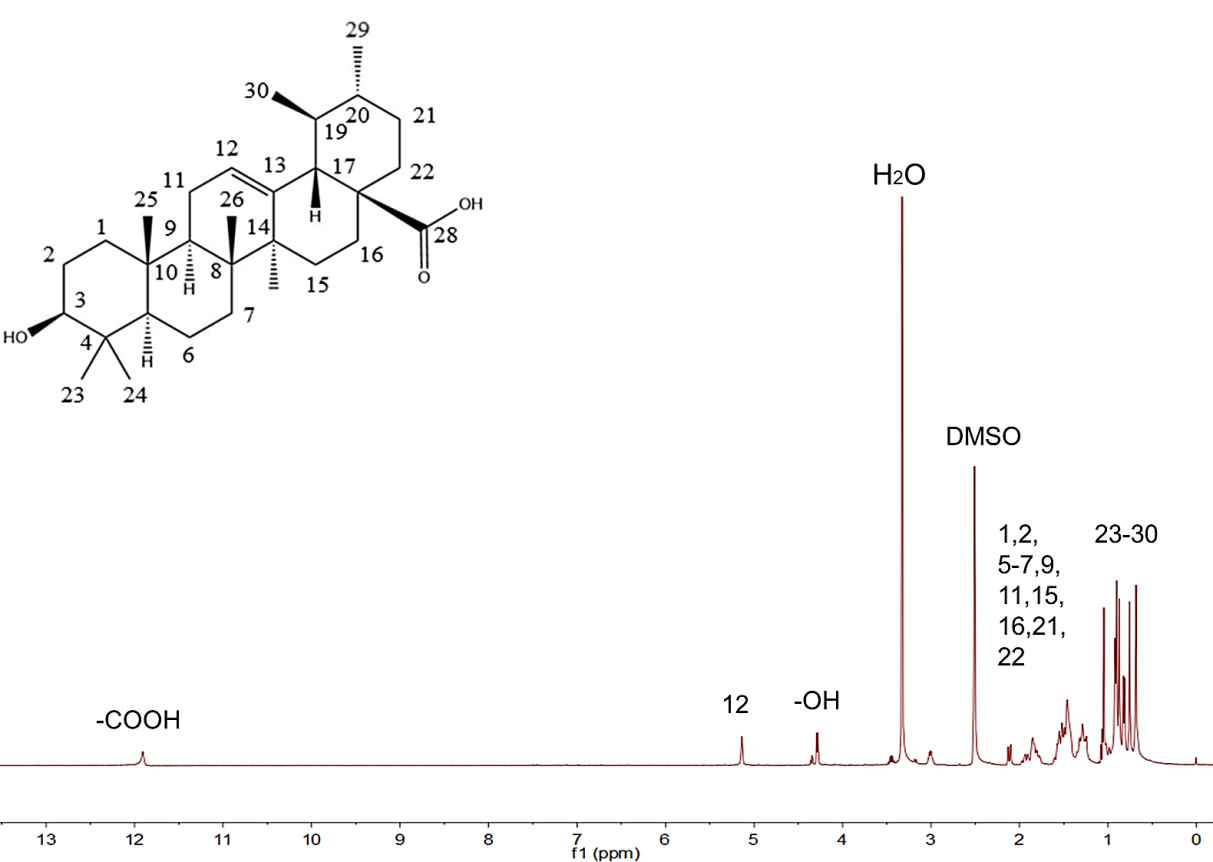


**Fig. S1** ^1^H-NMR spectra of UA in DMSO-d6.


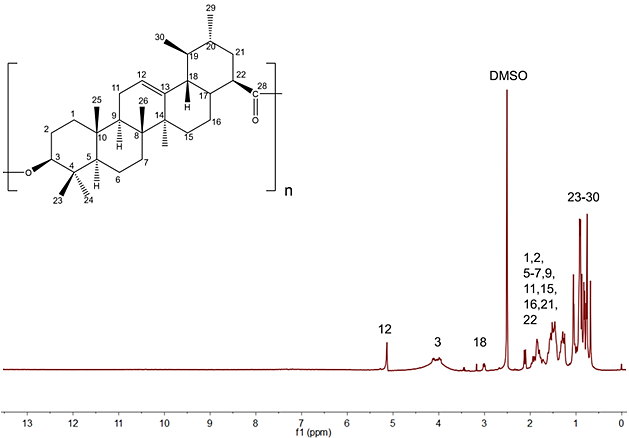


**Fig. S2** ^1^H-NMR spectra of PUA polymer in DMSO-d6.


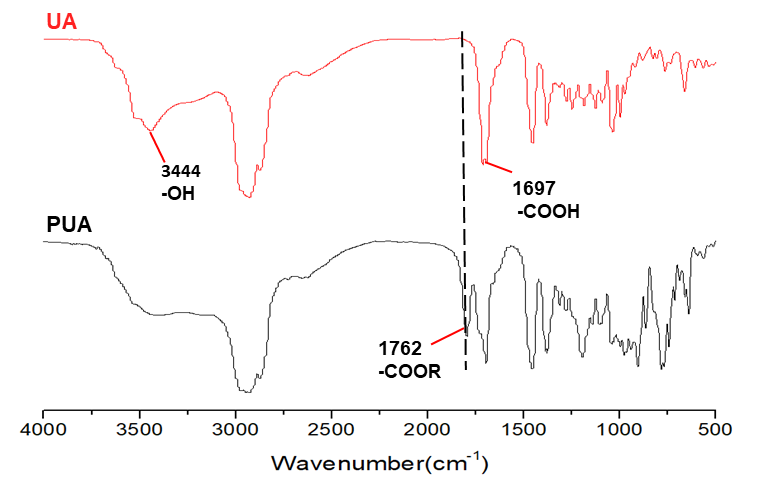


Fig. S3 FT-IR spectra of PUA and UA monomer.





**Fig.** **S4** TEM image of PUA NPs.


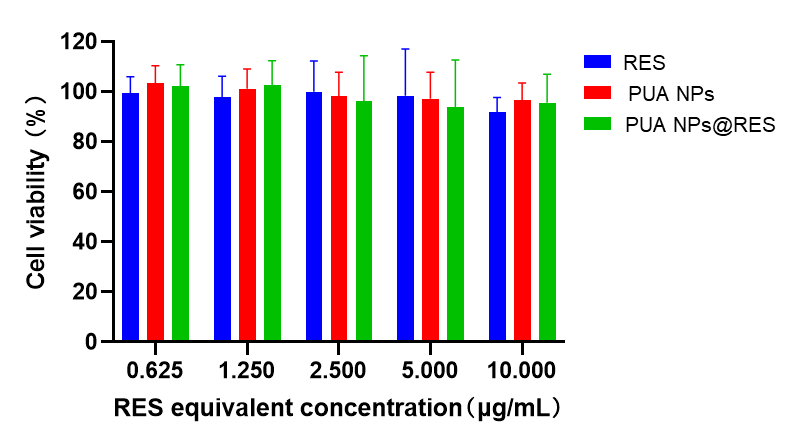


**Fig. S5** Cell viability of HK-2 cells treated free RES, PUA NPs and PUA NPs@RES across various equivalent RES concentrations for 24 h.


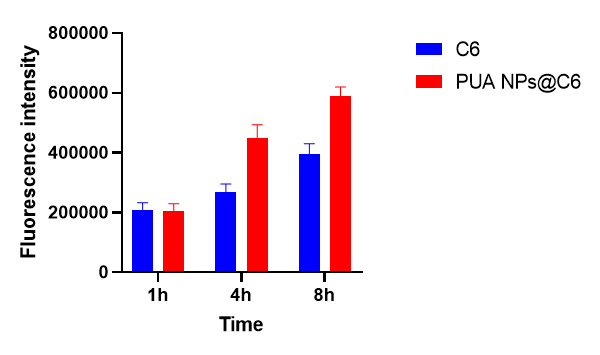


**Fig. S6** Quantitative analysis of cellular uptake of H_2_O_2_-stimulated HK-2 cells detected by flow cytometry.


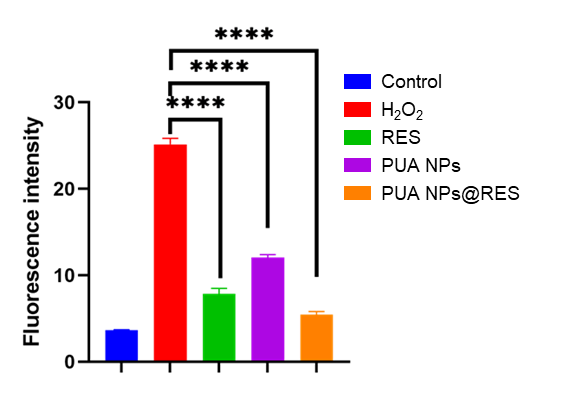


**Fig. S7** Quantitative analysis of intracellular ROS levels of H_2_O_2_-stimulated HK-2 cells detected by flow cytometry.


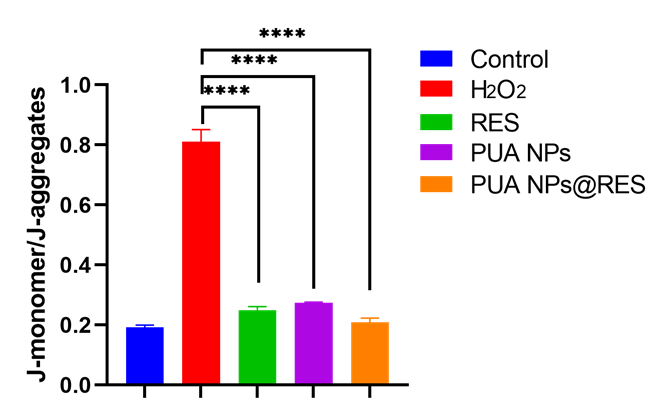


**Fig. S8** Quantitative analysis of mitochondrial membrane potential of H_2_O_2_-stimulated HK-2 cells after different treatments.


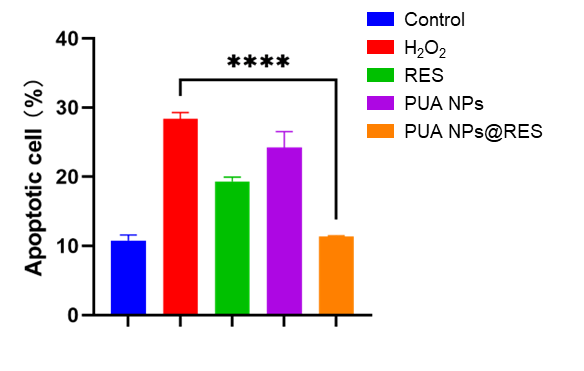


**Fig. S9** Florescence analysis of Cell apoptosis after different treatments.


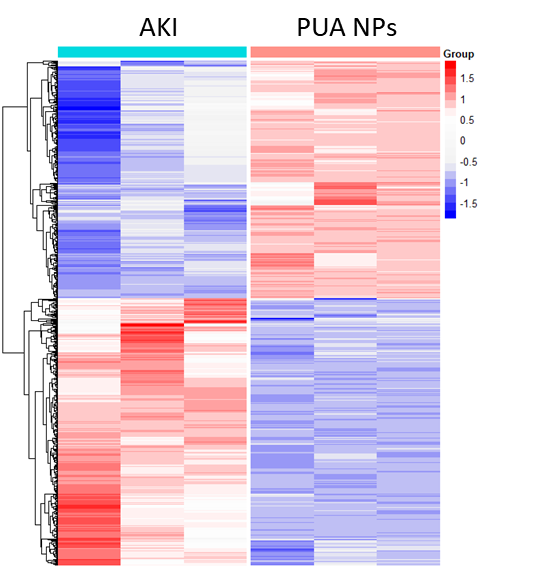


**Fig.S10** Heat map results of upregulated and downregulated genes after PUA NPs treatment (fold change ≥2 and P < 0.05).


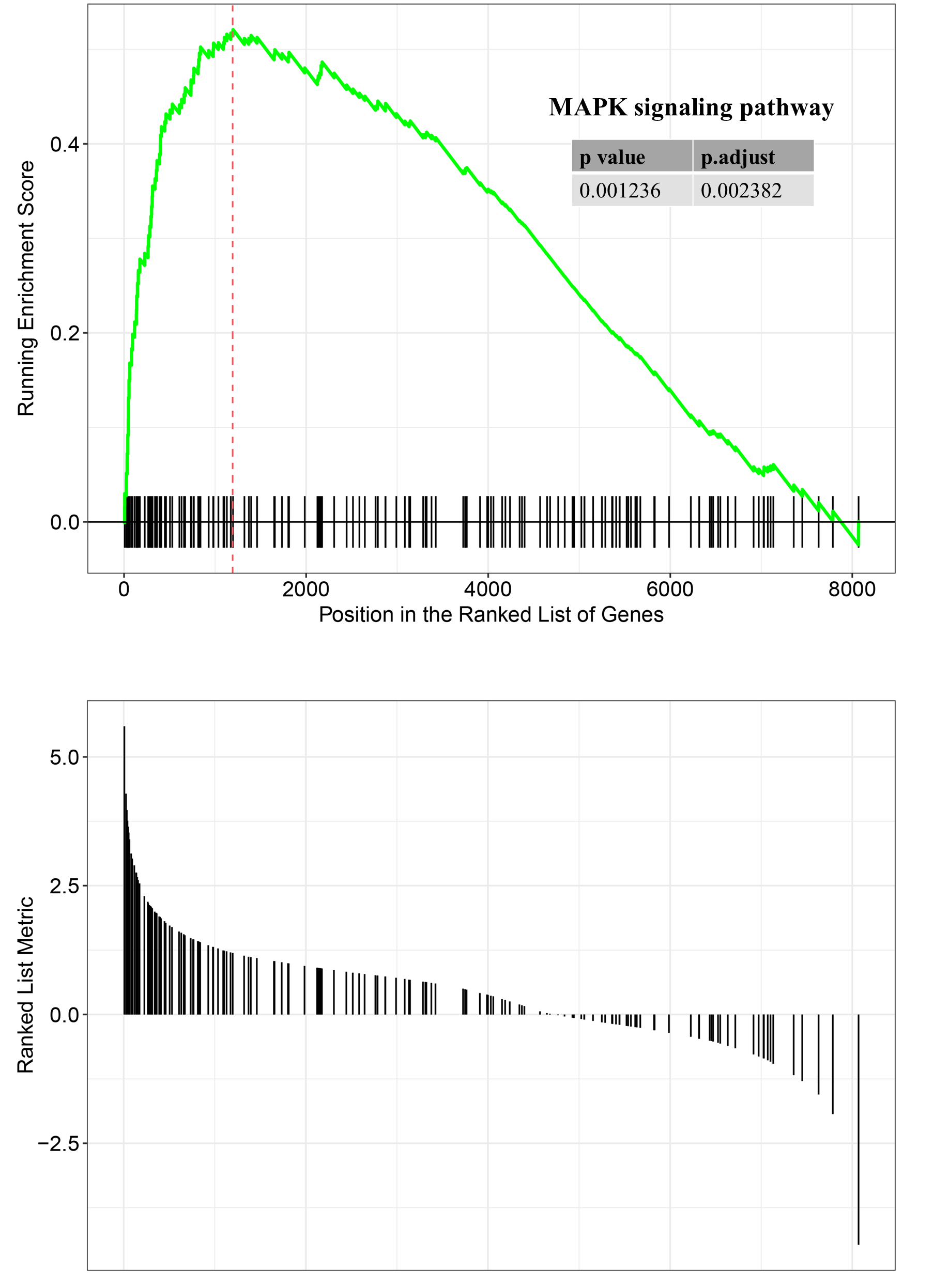


**Fig. S11** GSEA enrichment plots of gene set involved in MAPK signaling pathway.


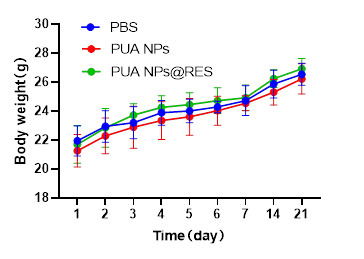


**Fig. S12** Changes in body weight of mice following with PBS, PUA NPs and PUA NPs@RES for 21 days.
